# Cardiovascular Risk Comparison between Expanded Hemodialysis Using Theranova and Online Hemodiafiltration (CARTOON): A Multicenter Randomized Controlled Trial

**DOI:** 10.1038/s41598-021-90311-6

**Published:** 2021-05-24

**Authors:** Yeonhee Lee, Myoung-jin Jang, Junseok Jeon, Jung Eun Lee, Wooseong Huh, Bum Soon Choi, Cheol Whee Park, Ho Jun Chin, Chae Lin Kang, Dong Ki Kim, Seung Seok Han, Kwon Wook Joo

**Affiliations:** 1 Department of Internal Medicine, Seoul National University College of Medicine, Seoul, South Korea; 2 Medical Research Collaborating Center, Seoul National University Hospital, Seoul, South Korea; 3 Division of Nephrology, Department of Medicine, Samsung Medical Center, Sungkyunkwan University School of Medicine, Seoul, South Korea; 4 Division of Nephrology, Department of Internal Medicine, College of Medicine, The Catholic University of Korea, Seoul, South Korea; 5 Department of Internal Medicine, Seoul National University Bundang Hospital, Gyeonggi-do, South Korea; 6 Department of Biomedical Sciences, Seoul National University College of Medicine, Seoul, South Korea

**Keywords:** Nephrology, Haemodialysis

## Abstract

Expanded hemodialysis (HDx) with medium cutoff (MCO) membranes, which remove middle-to-large molecules well, may be a good option to replace online hemodiafiltration (online-HDF). To provide more evidence, this randomized controlled trial compared several cardiovascular parameters between patients undergoing HDx and online-HDF. Eighty patients undergoing thrice-weekly hemodialysis were randomly assigned to receive either HDx with a Theranova membrane (n = 43) or online-HDF (n = 37). The primary endpoints were changes in brachial-ankle pulse wave velocity (baPWV), echocardiographic parameters, and coronary artery calcium (CAC) scores over 1 year, and the secondary endpoints included blood cardiovascular biomarkers, mortality, and patient-reported outcomes. A linear mixed model and log-rank test were used to estimate the group differences. 65 patients had completed the trial. The changes in baPWV and echocardiographic parameters did not differ between the two groups. The CAC scores remained stable in the online-HDF group, whereas an increasing trend was shown in the HDx group (*P* = 0.012). Other endpoints, including cardiovascular and all-cause mortalities, were similar between the two groups. The changes in cardiovascular parameters did not differ between HDx with an MCO membrane and online-HDF. However, attention may be needed in patients with high CAC scores or scores with an increasing tendency when online-HDF is replaced with HDx with an MCO membrane.

## Introduction

Cardiovascular disease is a leading cause of morbidity and mortality in patients with end-stage renal disease (ESRD)^1^. In addition to traditional cardiovascular risk factors, nontraditional factors, including uremic toxins, electrolyte and fluid imbalance, anemia, and inflammation, contribute to worsening cardiovascular outcomes in ESRD^2–5^. In particular, uremic toxins induce oxidative stress and vascular inflammation and remodeling^6–9^. Furthermore, uremic conditions induce platelet activation and aggregation, which leads to thrombus formation^7,8^. All of these factors can explain the high risk of cardiovascular diseases in patients with ESRD.

Uremic toxins are removed by hemodialysis (HD), but diffusion-based approaches in the conventional mode are limited in their ability to completely remove these toxins^10^. Given the potential association of middle-to-large uremic molecules but not small molecules with a high risk of cardiovascular disease^11,12^, the goal of treatment has been to enhance removal of these uremic toxins via HD membranes with increased permeability^13,14^. The technique used to increase the removal rate of middle-to-large molecules includes the use of high-flux membranes plus high dialysis intensity and alternative hemofiltration^15^. Although high-flux membranes improve the clearance of smaller middle molecules (e.g., β2-microglobulin), larger middle solutes (e.g., free light chains, most cytokines) may not be the target^10^. An increase in dialysis intensity has less effect on the plasma levels of potential cardiovascular toxins, which ultimately cannot improve patient survivals^16,17^. Online hemodialfiltration (online-HDF) is a great option for removing middle-to-large molecules with the help of ultrafiltration and subsequent convection. The online-HDF seems to represent the gold standard in removing uremic toxins among hemodialysis types^18^. This dialysis technique was related to improved cardiovascular outcomes in some studies^19,20^, although others did not show cardiovascular benefits related to online-HDF^21–26^. Despite the enhanced removal of middle-to-large molecules, it is difficult to make wide use of online-HDF due to the high cost, technical burden, and simultaneous risk of albumin loss^15^.

Expanded HD (HDx) is a treatment in which diffusion and convection are integrated inside a dialyzer equipped with a medium cutoff (MCO) membrane^27^. This novel HD method has shown greater removal of middle-to-large molecules than conventional HD^28–31^. However, it is unknown whether the use of MCO membranes confers cardiovascular benefits compared with other dialysis techniques. Because online-HDF was associated with improved cardiovascular survival compared with a high-flux dialyzer in some pooled analyses^19,20^, this randomized, controlled study tried to address the noninferiority of HDx with an MCO membrane to online-HDF in terms of cardiovascular outcomes. For this purpose, several cardiovascular parameters, such as brachial-ankle pulse wave velocity (baPWV), echocardiography, and blood markers, were traced over a one-year study period.

## Methods

### Study design

The study was a multicenter, prospective, open-label, randomized trial with prevalent HD patients from the dialysis units of four tertiary referral hospitals in South Korea. The study was approved by the review boards of all hospitals (Samsung Medical Center, 2018-04-047; Seoul St. Mary’s Hospital, KC18DEDV0209; Seoul National University Bundang Hospital, B-1804-463-401; and Seoul National University Hospital, D-1711-065-899) and was conducted in accordance with the principles of the Declaration of Helsinki. The study was registered at Clinical Research Information Service (https://cris.nih.go.kr/cris; ID, KCT0003188; date of registration, September 19th, 2018). The protocol was registered at Clinicaltrials.gov (ID: NCT03448887). All patients were recruited between April 2018 and May 2019 and signed an informed consent form to participate in the study. No changes were made to the procedures or study outcomes after trial commencement.

### Patients

Patients who underwent thrice-weekly in-center maintenance HD for > 3 months were eligible for the study if they met the following criteria: age 18 years old or older, receiving HD with a high-flux dialyzer for at least 1 month before enrollment, and providing informed consent. The exclusion criteria were as follows: receiving online-HDF or using MCO membranes before study enrollment, undergoing HD more or less than three times per week, having concurrent peritoneal dialysis, planning kidney transplantation within 1 year, having advanced or active cancer, having monoclonal and polyclonal gammopathies, being pregnant or planning to be pregnant, and being enrolled in other clinical trials.

### Treatment procedures

Patients were randomly assigned in a 1:1 ratio to receive HDx with a Theranova membrane (Theranova 400; Baxter International Inc., Hechingen, Germany) in the study group or online-HDF (Artis Physio system; Baxter International Inc.) with a high-flux dialyzer (Polyflux 170H or 210H dialyzer; Baxter International Inc.) in the control group using a web-based randomization system. Randomization was stratified by the participating center and patient age (≥ 65 or < 65 years old). HD was conducted in three 4-h sessions per week, and the postdilution volume-controlled mode with a target convective ultrafiltration volume of ≥ 19 L and a dialysate flow rate of ≥ 500 mL/min was used in online-HDF. Adverse events during the study period were noted whether or not they were related to the treatments.

### Data collection

Baseline clinical information was collected, such as age, sex, dialysis vintage, type of vascular access, comorbidities including diabetes mellitus, and previous history of myocardial infarction, cerebrovascular disease, congestive heart failure, peripheral vascular disease, and kidney transplantation. Kt/V was calculated by the second-generation formula for single-pool values to determine the appropriate level of HD.

All cardiovascular parameters were recorded at baseline and 6 and 12 months after enrollment. The primary endpoints were the changes in cardiovascular parameters, such as baPWV, echocardiographic parameters, and coronary artery calcium (CAC) scores, from baseline to 12 months after enrollment. baPWV was measured using a noninvasive vascular testing device (VP-1000 plus; Colin Co. Ltd., Japan). The values were obtained from the arm contralateral to the patient’s vascular access. Transthoracic two-dimensional echocardiography was conducted to estimate the left ventricular ejection fraction (LVEF) and left ventricular mass index (LVMI)^32^. Using pulsed-wave spectral Doppler tissue images, the early transmitral inflow (E) and early diastolic mitral annular peak (e′) velocities were calculated to display E/e′ as an indicator of LV diastolic function. Noncontrast cardiac computed tomography was performed to obtain the CAC score. The Agatston score was used to calculate the CAC scores, which ranged from 0 to several thousand, indicating extensive coronary atherosclerosis^33^. The CAC scores were available in 41 patients in the HDx group and 35 patients in the online-HDF group, because 4 patients had stents in coronary arteries. Blood cardiovascular biomarkers, such as troponin I and T, brain natriuretic peptide (BNP), and N-terminal prohormone of BNP (NT-proBNP), were measured using electrochemiluminescence immunoassays. Plasma interleukin (IL)-6 was measured using an enzyme-linked immunosorbent assay kit (R&D Systems, Inc., Minneapolis, MN, USA). Other blood markers, such as albumin, calcium, phosphate, and high-sensitivity C-reactive protein, were also recorded by immunoturbidimetry and colorimetric assays.

Patient-reported outcomes, such as the Dialysis Symptom Index (DSI)^34^, the degree of fatigue^35^, and the recovery time after dialysis^36^, were collected as secondary endpoints. The DSI contained 30 items targeting specific and common physical and emotional symptoms of HD patients. The degree of fatigue after dialysis ranged from 0 to 10, with high degrees indicating worse fatigue. The patients were requested to respond to a single open-ended question, “How long does it take you to recover from a dialysis session?” The recovery time was scored at five levels as follows: 1, within minutes; 2, upon arriving home; 3, by bedtime; 4, by the next morning; and 5, by the next dialysis session. Mortality and its causes were also evaluated based on prospective monitoring.

### Statistical analysis

The sample size was driven by the primary endpoint, baPWV. When standard deviations of 0.5 m/s and 0.4 m/s in the online-HDF and conventional HD, respectively, from the best estimates were assumed^37^, a total of 74 patients were needed to provide 80% power at a two-sided alpha level of 0.05, to detect a mean difference of 0.3 m/s between the effect of the two modalities. We had added approximately 10% more participants to account for potential dropouts, resulting in a final enrollment goal of 80 participants (40 per group).

All analyses were conducted based on the intention-to-treat principle. Categorical and continuous variables are expressed as proportions and the means ± standard deviations for normally distributed variables and as the median with interquartile range for nonnormally distributed variables. A comparison of baseline characteristics was performed with the unpaired t-test or Mann–Whitney *U* test for continuous variables and with the chi-square test for categorical variables. Linear mixed-effects models were used to assess changes from baseline and differences between groups in primary and secondary endpoints with treatment assignment and time as fixed effects and patients as random effects. The estimated changes at 6 and 12 months in each group and the differences between groups (i.e., the HDx group relative to the online-HDF group) are presented with means and 95% confidence intervals. Because there was no prespecified plan to adjust for multiple comparisons, *P* values and their corresponding 95% confidence intervals were not adjusted for multiple tests, and a Bonferroni-adjusted significance of 0.025 (i.e., 0.05 divided by two) was applied to account for two tests for between-group differences at 6 and 12 months. Kaplan–Meier survival curves were drawn to evaluate the all-cause and cardiovascular mortalities. The log-rank test was used to compare survival curves. Cox proportional hazard ratio models were conducted to calculate hazard ratios of the mortality risk. A *P* value less than 0.05 was considered significant in other analyses. All of the analyses were performed using STATA software (version 16.1; StataCorp LP, College Station, TX, USA) and GraphPad Prism (version 9.0.2; GraphPad Software, Inc., La Jolla, CA, USA).

## Results

### Baseline characteristics of patients

**Figure 1:**
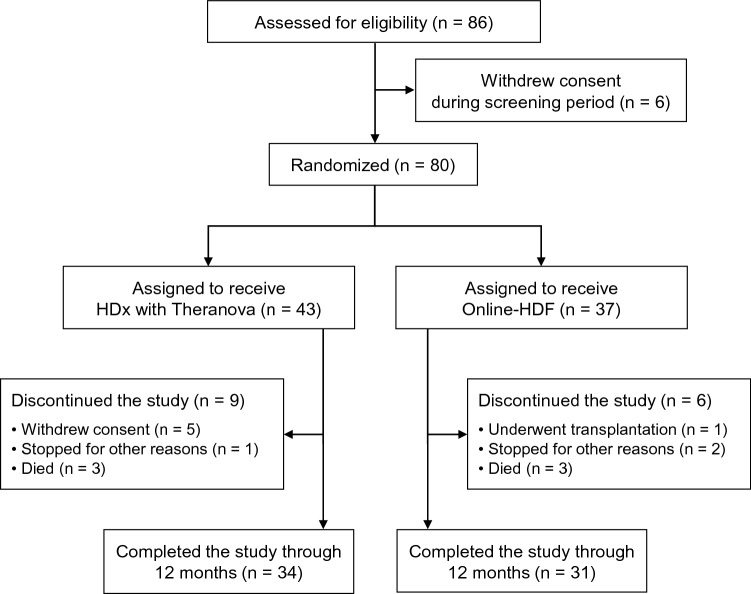
Flow chart of study patients. *HDx* expanded hemodialysis, *HDF* hemodiafiltration.

Eighty-six patients were initially assessed for eligibility for the study between April 2018 and May 2019. Six patients were excluded because they refused to participate in the study. Finally, a total of 80 patients were randomized, and 65 patients were followed up until the end of the study (Fig. 1). Among 5 patients who withdrew consents in the HDx group after starting trial, 2 patients refused to receive tests, 2 patients switched to other hemodialysis modes due to patient need, and 1 patient was transferred to another center. There was no withdrawal of consent in the online-HDF group after starting trial. The mean age of all patients was 62 ± 14 years old, and 58.8% were men. The mean Kt/V value was 1.71 ± 0.33. The mean value of achieved convective volume adjusted by weight was 0.3 ± 0.1 L/kg/session in the online-HDF group. Other baseline characteristics are shown in Table 1. These baseline characteristics did not differ between the two groups.

**Table 1:** Baseline characteristics of the study subjects.

	All (n = 80)	HDx (n = 43)	Online-HDF (n = 37)	*P* value
Age (years)	61.8 ± 14.0	62.9 ± 11.6	60.6 ± 16.4	0.484
Male (%)	58.8	51.2	67.6	0.137
Body mass index (kg/m^2^)	22.1 ± 3.4	22.4 ± 3.7	21.8 ± 3.0	0.457
Time on dialysis (months)	46 (16–78)	40 (14–72)	50 (40–93)	0.075
**Vascular access (%)**				0.823
Fistula	90.0	90.7	89.2	
Graft	10.0	9.3	10.8	
Diabetes mellitus (%)	45.0	46.5	43.2	0.770
**Previous cardiovascular disease (%)**				
Myocardial infarction	23.8	27.9	18.9	0.346
Cerebrovascular disease	12.5	7.0	18.9	0.107
Congestive heart failure	11.3	11.6	10.8	0.908
Peripheral vascular disease	18.8	20.9	16.2	0.590
History of kidney transplantation	2.5	4.7	0	0.184
Calcium-based phosphate binder use (%)	25.0	20.9	29.7	0.365
Non-calcium-based phosphate binder use (%)	67.5	72.1	62.2	0.344
spKt/V	1.71 ± 0.33	1.71 ± 0.26	1.71 ± 0.39	0.992
Hemoglobin (g/dL)	11.1 ± 1.0	11.2 ± 1.0	11.0 ± 1.0	0.339
Albumin (g/dL)	3.9 ± 0.3	3.9 ± 0.3	3.9 ± 0.3	0.844
Calcium (mg/dL)	9.0 ± 0.6	8.9 ± 0.6	9.0 ± 0.6	0.649
Phosphorus (mg/dL)	4.7 ± 1.3	4.6 ± 1.4	4.7 ± 1.3	0.683

Data are expressed as the means ± standard deviations, medians (interquartile range), or proportions, as appropriate.

*HDx* expanded hemodialysis, *HDF* hemodiafiltration.

### Cardiovascular parameters

At baseline, the cardiovascular parameters did not differ between the two groups (Table 2). The changes in baPWV and echocardiographic parameters such as LVEF, LVMI, and E/e′ were not different between the two groups (Table 2). Figure S1A,B, which show the trends of the parameters over the study period, support these results. The CAC scores remained stable in the online-HDF group, whereas an increasing trend was shown in the HDx group (Table 2, Fig. S1C). When some of the baseline characteristics that had a *P* value of < 0.1 in the comparison analysis (e.g., sex, diabetes mellitus, previous history of cardiovascular disease, and dialysis vintage) were further adjusted, the overall results remained unaltered (Table S1). As a reference, the baseline values of and changes in calcium and phosphate did not differ between the two groups (Table S2).

**Table 2:** Linear mixed-effects model for the change in cardiovascular biomarkers.

Variables	Baseline values	Change from the baseline (mean and 95% confidence intervals)			
		6 months	*P* value	12 months	*P* value
**baPWV (m/s)**					
HDx	1.8 ± 0.7	0.1 (0 to 0.2)	0.176	0 (− 0.1 to 0.2)	0.518
Online-HDF	1.9 ± 0.7	− 0.1 (− 0.2 to 0)	0.221	0.1 (0 to 0.3)	0.046
Between-group difference		0.2 (0 to 0.3)	0.066	− 0.1 (− 0.3 to 0.1)	0.317
**LVEF (%)**					
HDx	63.0 (57.0–69.0)	− 0.2 (− 2.5 to 2.1)	0.860	− 0.7 (− 3.1 to 1.7)	0.561
Online-HDF	63.0 (56.0–66.0)	0.6 (–1.8 to 3.0)	0.622	− 0.6 (− 3.1 to 1.9)	0.660
Between-group difference		− 0.8 (− 4.1 to 2.6)	0.648	− 0.1 (− 3.5 to 3.4)	0.966
**LVMI (g/m**^**2**^**)**					
HDx	111.3 (88.7–138.9)	− 36.1 (− 79.9 to 7.6)	0.106	− 39.2 (− 84.0 to 5.5)	0.086
Online-HDF	114.7 (102.2–142.9)	− 22.5 (− 69.8 to 24.8)	0.351	− 54.7 (− 103.2 to − 6.1)	0.027
Between-group difference		− 13.4 (− 77.7 to 50.8)	0.682	14.5 (− 51.4 to 80.4)	0.666
**E/e**′					
HDx	12.0 (10.0–16.0)	0.3 (− 1.0 to 1.7)	0.628	0.4 (− 1.0 to 1.8)	0.536
Online-HDF	12.8 (10.0–14.8)	− 0.8 (− 2.3 to 0.6)	0.274	− 0.2 (− 1.7 to 1.3)	0.815
Between-group difference		1.2 (− 0.8 to 3.2)	0.240	0.7 (− 1.4 to 2.7)	0.516
Coronary artery calcium score*					
HDx	295 (19–799)	64.6 (0.7 to 128.5)	0.048	154.9 (91.0 to 218.8)	< 0.001
Online-HDF	268 (16–678)	23.0 (− 43.1 to 89.1)	0.496	36.6 (− 29.5 to 102.7)	0.277
Between-group difference		41.6 (− 50.3 to 133.6)	0.375	118.3 (26.3 to 210.2)	0.012

*baPWV* brachial-ankle pulse wave velocity, *HDx* expanded hemodialysis, *HDF* hemodiafiltration, *LVEF* left ventricular ejection fraction, *LVMI* left ventricular mass index, *E* peak early mitral inflow velocity, *e*′ peak early diastolic mitral annular velocity.

*Available for 41 of the HDx group patients and 35 of the online-HDF group patients.

### Blood cardiovascular biomarkers

The changes in blood biomarkers, including troponins I and T, BNP, NT-proBNP, high-sensitivity C-reactive protein, and IL-6, did not differ between the two groups (Table 3). When additional factors were adjusted, the overall results remained unaltered (Table S1). The change in albumin levels over the study period did not differ between the two groups, with between-group differences of − 0.06 (− 0.20 to 0.09) at 6 months and − 0.10 (− 0.25 to 0.05) at 12 months (*P*s > 0.05).

**Table 3:** Linear mixed-effects model for the change in blood biomarkers.

Variables	Baseline values	Change from the baseline (mean and 95% confidence intervals)			
		6 months	*P* value	12 months	*P* value
**BNP (pg/mL)**					
HDx	369.1 (185.0–910.0)	51.4 (− 267.9 to 370.7)	0.752	127.4 (− 206.1 to 460.9)	0.454
Online-HDF	287.2 (139.5–907.0)	− 140.7 (− 487.4 to 205.9)	0.426	− 111.7 (− 470.0 to 246.7)	0.541
Between-group difference		192.2 (− 279.2 to 663.5)	0.424	239.1 (− 250.4 to 728.6)	0.338
**NT-proBNP (ng/mL)**					
HDx	3.95 (2.16–6.83)	− 0.31 (− 4.19 to 3.56)	0.874	1.42 (− 2.64 to 5.48)	0.493
Online-HDF	4.27 (2.19–16.82)	− 3.24 (− 7.40 to 0.92)	0.127	− 3.32 (− 7.61 to 0.98)	0.130
Between-group difference		2.96 (− 2.72 to 8.64)	0.307	4.66 (− 1.25 to 10.57)	0.122
**Troponin-I (ng/mL)**					
HDx	0.06 (0.04–0.09)	0 (0 to 0.01)	0.538	0 (− 0.01 to 0.01)	0.550
Online-HDF	0.06 (0.03–0.07)	0 (− 0.01 to 0.01)	0.901	0 (0 to 0.01)	0.189
Between-group difference		0 (− 0.03 to 0.01)	0.224	0 (− 0.02 to 0.02)	0.926
**Troponin-T (ng/mL)**					
HDx	0.06 (0.04–0.09)	0 (− 0.01 to 0)	0.362	0 (− 0.01 to 0.01)	0.437
Online-HDF	0.06 (0.03–0.07)	− 0.01 (− 0.02 to 0)	0.130	− 0.01 (− 0.02 to 0)	0.139
Between-group difference		0 (− 0.01 to 0.02)	0.595	0 (− 0.01 to 0.02)	0.526
**C-reactive protein (mg/dL)**					
HDx	0.07 (0.04–0.23)	0.21 (− 0.30 to 0.72)	0.421	0.10 (− 0.43 to 0.62)	0.722
Online-HDF	0.09 (0.04–0.23)	0.47 (− 0.08 to 1.01)	0.095	− 0.02 (− 0.54 to 0.58)	0.951
Between-group difference		− 0.25 (− 1.00 to 0.50)	0.511	0.08 (− 0.69 to 0.74)	0.835
**Interleukin-6**					
HDx	9.55 (7.78–11.48)	4.09 (− 2.28 to 10.47)	0.208	1.85 (− 4.76 to 8.47)	0.582
Online-HDF	8.40 (7.02–11.94)	0.55 (− 6.17 to 7.27)	0.872	− 1.88 (− 8.77 to 5.00)	0.592
Between-group difference		3.54 (− 5.72 to 12.80)	0.453	3.74 (− 5.81 to 13.28)	0.443

*BNP* brain natriuretic peptide, *HDx* expanded hemodialysis, *HDF* hemodiafiltration, *NT-proBNP* N-terminal prohormone of brain natriuretic peptide.

### All-cause and cardiovascular mortality

**Figure 2:**
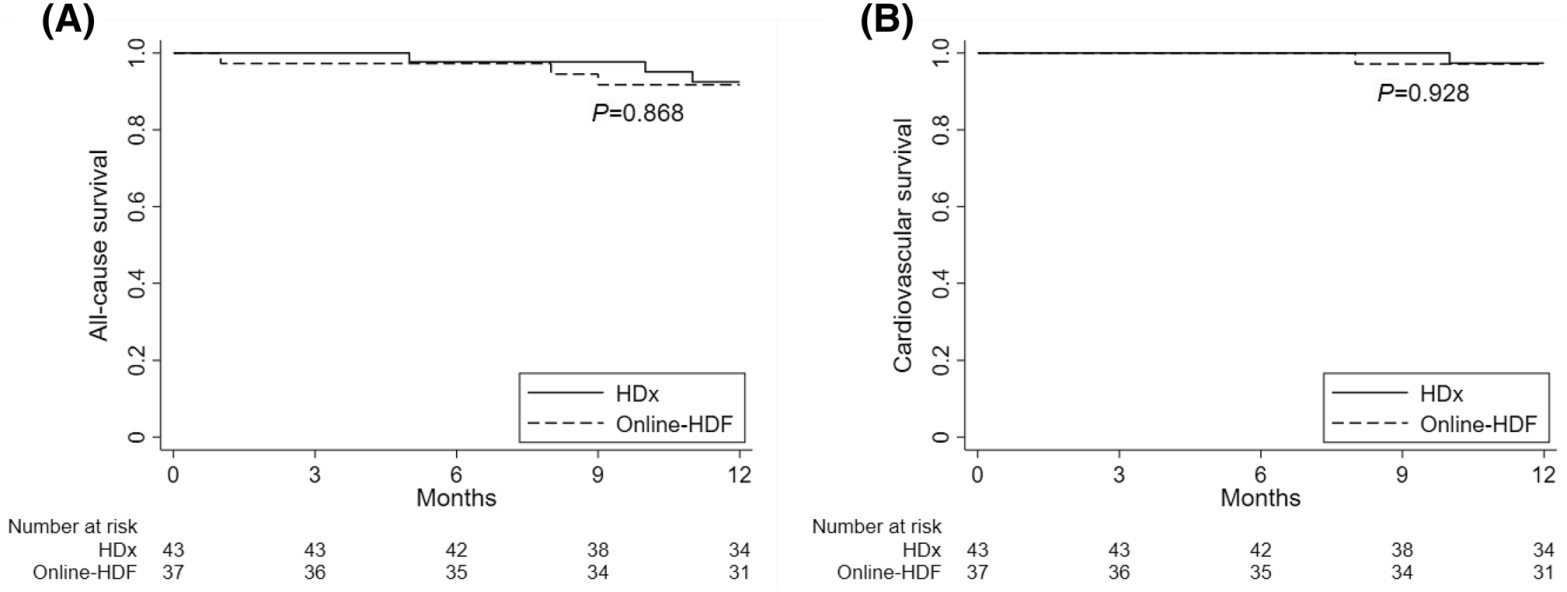
Kaplan–Meier curves of all-cause (**A**) and cardiovascular (**B**) survival. *HDx* expanded hemodialysis, *HDF* hemodiafiltration.

There were 6 deaths (7.5%; 3 in the HDx group and 3 in the online-HDF group) during the study period (Table S3). Of them, 2 patients died due to cardiovascular events. As shown in Fig. 2, the survival curves for cardiovascular and all-cause mortality did not differ between the two groups. When the Cox models were applied, the HDx and online-HDF groups had similar risks of cardiovascular and all-cause mortality (Table S4).

### Patient-reported outcome

The baseline DSI scores, degree of fatigue, and recovery time after dialysis were not different between the two groups (Table 4). The change trends in these endpoints did not differ between the two groups.

**Table 4:** Linear mixed-effects model for the change in patient-reported outcomes.

Variables	Baseline values	Change from the baseline (mean and 95% confidence intervals)			
		6 months	*P* value	12 months	*P* value
**Number of symptoms in DSI**					
HDx	12 (5–20)	− 2.0 (− 3.8 to − 0.3)	0.024	− 1.4 (− 3.3 to 0.5)	0.140
Online-HDF	11 (4–16)	− 0.5 (− 2.3 to 1.4)	0.634	− 1.0 (− 2.9 to 1.0)	0.321
Between-group difference		− 1.6 (− 4.2 to 1.0)	0.231	− 0.4 (− 3.1 to 2.3)	0.765
**Overall symptom severity score in DSI**					
HDx	17 (10–37)	− 2.1 (–6.1 to 1.9)	0.299	− 2.7 (− 6.8 to 1.5)	0.208
Online-HDF	18 (6–27)	− 1.4 (–5.6 to 2.8)	0.517	− 1.3 (− 5.7 to 3.1)	0.567
Between-group difference		− 0.7 (− 6.5 to 5.1)	0.814	− 1.4 (− 7.4 to 4.6)	0.651
**Degree of fatigue after dialysis**					
HDx	6 (5–7)	0.1 (− 0.6 to 0.9)	0.702	− 0.6 (− 1.3 to 0.2)	0.164
Online-HDF	5 (3–6)	− 0.3 (− 1.1 to 0.5)	0.513	0.1 (− 0.8 to 0.9)	0.880
Between-group difference		0.4 (− 0.7 to 1.5)	0.459	− 0.6 (− 1.8 to 0.5)	0.287
**Recovery time after dialysis**					
HDx	3 (2–3)	0 (− 0.3 to 0.3)	0.975	0 (− 0.3 to 0.4)	0.786
Online-HDF	2 (1–3)	− 0.1 (− 0.4 to 0.3)	0.730	− 0.1 (− 0.4 to 0.3)	0.713
Between-group difference		0 (− 0.4 to 0.5)	0.912	0.1 (− 0.4 to 0.6)	0.644

*DSI* dialysis symptom index, *HDx* expanded hemodialysis, *HDF* hemodiafiltration.

## Discussion

Uremic toxin, a result of ESRD, is associated with high cardiovascular risk. However, conventional HD may not be enough to remove all uremic toxins, particularly middle-to-large molecules. This prospective, open-label, multicenter, randomized, controlled trial indicated that HDx with an MCO membrane was not inferior to online-HDF in terms of cardiovascular risk according to the change trends in the values of baPWV, LVEF, LVMI, E/e′, and other blood biomarkers. Additionally, the changes in patient-reported outcomes did not differ between the two groups. However, compared with the online-HDF group, the HDx group had an increasing trend in CAC scores, although this endpoint was not followed in some patients.

Previous studies have shown the superiority of HDx with a Theranova membrane to conventional HD in removing middle-to-large molecules while ensuring retention of albumin^14,28–31,38^. A representative randomized, controlled trial wherein 86 patients received HDx with a Theranova membrane and 86 other patients received conventional high-flux dialyzer identified a great reduction in middle-to-large molecules such as free light chains, complements, and cytokines when using HDx, but serum albumin levels were maintained similarly between the two groups^30^. However, there are no studies that address the effects of HDx on cardiovascular endpoints, such as surrogate markers and mortality, and this clinical trial was the first to compare cardiovascular parameters between HDx and online-HDF.

Five randomized, controlled trials have been conducted in different European countries to determine the benefits for survival and other outcomes (e.g., cardiovascular events, blood biomarkers, and quality of life) in online-HDF^22–26^. Of them, the Estudio de Supervivencia de Hemodiafiltración On-Line trial was the only one to show a reduction in all-cause and cardiovascular mortalities for the online-HDF arm compared with the high-flux HD arm. None of the others showed elevated cardiovascular survival, although the clearance of middle-to-large molecules improved in online-HDF. Corresponding meta-analyses reported survival and cardiovascular benefits for online-HDF compared with conventional HD^20,21,39–41^. Post hoc and pooled analyses suggest that a high convection volume may be needed to show better outcomes in online-HDF than in conventional HD^22,23,25^. European regulatory authorities have allowed online-HDF to be used in patients with uncontrolled hyperphosphatemia and those suffering from complications such as amyloidosis^21,42^, but this issue may not be similarly accepted in other countries because of limitations in setting up online-HDF. Before conducting online-HDF, new machines, quality control in substituting fluids, and trained staff are needed, all of which limit its utilization, particularly in primary clinics or developing countries^43^. The present results may support the interchangeability of HDx with an MCO membrane when online-HDF is recommended in terms of cardiovascular benefit.

In terms of uremic status, vascular remodeling, characterized by a high degree of both intimal and medial calcifications, is promoted via bone-related proteins and transformation of vascular smooth muscle cells into osteoblastic-like cells^7,9,44^. The CAC, a marker of vascular remodeling in ESRD patients^45,46^, seemed to be worsening in the HDx group compared with the online-HDF group. However, not only were these results based on subgroup analysis because of missing data in some patients, but the normal aging process might mask the changes caused by uremia-related calcification, particularly in elderly patients^9^. Nevertheless, the choice of HD modality between HDx with an MCO membrane and online-HDF may be affected, particularly when CAC scores are aggravated along with HD.

Previous studies have shown that patients on online-HDF had better quality of life than patients on conventional HD^47,48^, but one randomized, controlled trial (n = 334 in high-flux HD; n = 347 in online-HDF) did not demonstrate a benefit of online-HDF using the Kidney Disease Quality of Life-Short form survey^49^. In the case of HDx with an MCO membrane, a randomized, controlled trial (n = 24 in HDx with a Theranova membrane; n = 25 in high-flux dialyzer) demonstrated the benefit of HDx in quality of life, although another randomized, controlled trial (n = 86 in HDx with a Theranova membrane; n = 86 in high-flux dialyzer) showed no difference between groups^50^. The present trial measured three different patient-reported outcomes, because these are more predictive of outcomes than the Kidney Disease Quality of Life-Short form^35,36,51,52^, and found no difference between HDx and online-HDF. Based on these results, HDx with an MCO membrane may be a good alternative to online-HDF in patients receiving conventional HD who want to use the latter in order to improve their quality of life.

Although the trial results are informative, there are some issues that need to be discussed. The trial was conducted only in Korean patients, and thus, the dialysis settings or the risk of cardiovascular disease may be different in other populations. The study duration and sample size were relatively short and low, respectively. Most of the measurements were conducted in the individual hospitals, not in a central laboratory, which might increase the measurement variability, although the same protocol was used. Other important parameters were not examined, such as residual kidney function, intradialytic hemodynamic stability, and nutritional status.

HDx with an MCO membrane was not inferior to online-HDF in terms of cardiovascular parameters. Accordingly, the former can be a good alternative to the latter. This issue may be more important in clinics where online-HDF is not available. However, when online-HDF is replaced with HDx with an MCO membrane, attention may be needed in patients with a high CAC score or a CAC score with an increasing trend. Large clinical trials with other populations and long-term follow-up durations are needed to confirm the present results.

## Supplementary Information

Supplementary Information.
