# Supplementary material for: Cardiovascular Risk Comparison between Expanded Hemodialysis Using Theranova and Online Hemodiafiltration (CARTOON): A Multicenter Randomized Controlled Trial
Source: Sci Rep. 2021 May 24;11:10807. doi: 10.1038/s41598-021-90311-6 (PMC8144214; doi:10.1038/s41598-021-90311-6)

**Supplementary data table of contents**

Supplemental Table S1. Multivariable-adjusted linear mixed-effects model.

Supplemental Table S2. Linear mixed-effects model for the changes in calcium and phosphate.

Supplemental Table S3. Number of mortality events.

Supplemental Table S4. Factors relevant to the risk of cardiovascular and all-cause mortality.

Supplemental Figure S1. Change in cardiovascular parameters over 12 months. (A), Brachial-ankle pulse wave velocity (baPWV). (B), Echocardiographic parameters. (C), Coronary artery calcium (CAC) score.

Supplemental Table S1. Multivariable-adjusted linear mixed-effects model

|  | Between-group difference* (mean and 95% confidence intervals) | | | |
| --- | --- | --- | --- | --- |
| Variables | 6 months | *P* value | 12 months | *P* value |
| Cardiovascular biomarkers |  |  |  |  |
| baPWV (m/s) | 0.2 (0 to 0.3) | 0.069 | –0.1 (–0.3 to 0.1) | 0.310 |
| LVEF (%) | –0.8 (–4.2 to 2.5) | 0.631 | –0.1 (–3.6 to 3.3) | 0.940 |
| LVMI (g/m^2^) | –13.6 (–78.0 to 50.8) | 0.679 | 15.4 (–50.6 to 81.5) | 0.647 |
| E/e’ | 1.1 (–0.8 to 3.1) | 0.259 | 0.6 (–1.4 to 2.7) | 0.552 |
| Coronary artery calcium score | 41.6 (–50.3 to 133.6) | 0.375 | 118.3 (26.3 to 210.2) | 0.012 |
| Blood biomarkers |  |  |  |  |
| BNP (pg/mL) | 192.5 (–278.9 to 663.9) | 0.423 | 253.6 (–235.8 to 743.0) | 0.338 |
| NT-proBNP (ng/mL) | 2.93 (–2.76 to 8.61) | 0.313 | 4.74 (–1.17 to 10.65) | 0.116 |
| Troponin I (ng/mL) | –0.01 (–0.03 to 0.01) | 0.217 | 0 (–0.02 to 0.02) | 0.981 |
| Troponin T (ng/mL) | 0 (–0.01 to 0.01) | 0.630 | 0 (–0.01 to 0.02) | 0.595 |
| C-reactive protein (mg/dL) | –0.26 (–1.00 to 0.49) | 0.499 | 0.08 (–0.69 to 0.85) | 0.843 |
| Interleukin-6 | 3.53 (–5.73 to 12.79) | 0.455 | 3.67 (–5.87 to 13.22) | 0.451 |

baPWV, brachial-ankle pulse wave velocity; LVEF, left ventricular ejection fraction; LVMI, left ventricular mass index; E, peak early mitral inflow velocity; e’, peak early diastolic mitral annular velocity; BNP, brain natriuretic peptide; NT-proBNP, N-terminal prohormone of brain natriuretic peptide.

*Adjusted for sex, diabetes mellitus, previous history of cardiovascular disease, and dialysis vintage.

Supplemental Table S2. Linear mixed-effects model for the changes in calcium and phosphate.

|  |  | Change from the baseline (mean and 95% confidence intervals) | | | |
| --- | --- | --- | --- | --- | --- |
| Variables | Baseline values | 6 months | *P* value | 12 months | *P* value |
| Calcium (mg/dL) |  |  |  |  |  |
| HDx | 8.9 ± 0.6 | –0.28 (–0.49 to –0.07) | 0.010 | –0.22 (–0.45 to 0.01) | 0.057 |
| Online-HDF | 9.0 ± 0.6 | –0.39 (–0.62 to –0.17) | 0.001 | –0.17 (–0.39 to 0.05) | 0.136 |
| Between-group difference |  | 0.12 (–0.19 to 0.42) | 0.455 | 0.06 (–0.26 to 0.38) | 0.723 |
| Phosphate (mg/dL) |  |  |  |  |  |
| HDx | 4.6 ± 1.4 | 0.22 (–0.27 to 0.72) | 0.370 | 0.27 (–0.24 to 0.78) | 0.303 |
| Online-HDF | 4.7 ± 1.3 | 0.23 (–0.63 to 0.42) | 0.696 | –0.26 (–0.80 to 0.29) | 0.352 |
| Between-group difference |  | 0.33 (–0.39 to 1.05) | 0.370 | 0.53 (–0.22 to 1.28) | 0.166 |

HDx, expanded hemodialysis; HDF, hemodiafiltration.

Supplemental Table S3. Number of mortality events

|  | HDx (n = 43) | | Online-HDF (n = 37) | |
| --- | --- | --- | --- | --- |
|  | Number | Time to events (months) | Number | Time to events (months) |
| All-cause mortality | 3 | 26 | 3 | 18 |
| Cardiovascular mortality | 1 | 10 | 1 | 8 |
| Infectious mortality | 0 | 0 | 1 | 9 |
| Other causes | 2 | 16 | 1 | 1 |

HDx, expanded hemodialysis; HDF, hemodiafiltration.

Supplemental Table S4. Factors relevant to the risk of cardiovascular and all-cause mortality

|  | Cardiovascular mortality | | All-cause mortality | |
| --- | --- | --- | --- | --- |
|  | HR (95% CI) | *P* value | HR (95% CI) | *P* value |
| HDx (vs. Online-HDF) | 0.881 (0.055–14.090) | 0.928 | 0.878 (0.177–4.350) | 0.873 |
| Age (per 1-year increase) | 1.069 (0.931–1.228) | 0.343 | 1.032 (0.965–1.104) | 0.355 |
| Female (vs. male) | 1.464 (0.092–23.425) | 0.787 | 0.732 (0.134–3.997) | 0.719 |
| Diabetes (vs. none) | 1.346 (0.084–21.536) | 0.834 | 1.302 (0.263–6.456) | 0.747 |
| Previous history of cardiovascular disease (vs. none) | 1.384 (0.087–22.130) | 0.818 | 1.396 (0.282–6.919) | 0.683 |
| Time on dialysis (per 1 month) | 0.993 (0.962–1.025) | 0.673 | 1.007 (0.998–1.015) | 0.124 |
| Graft (vs. fistula) | N/A* |  | 1.959 (0.228–16.793) | 0.540 |

HR, hazard ratio; CI, confidence interval; HDx, expanded hemodialysis; HDF, hemodiafiltration; N/A, not available.

*There were no events in the group with grafts.

Supplemental Figure S1. Change in cardiovascular parameters over 12 months. (A), Brachial-ankle pulse wave velocity (baPWV). (B), Echocardiographic parameters. (C), Coronary artery calcium (CAC) score. Means and 95% confidence intervals are presented. The blue circles and red squares indicate the expanded hemodialysis with a Theranova membrane and online hemodiafiltration groups, respectively. LVEF, left ventricular ejection fraction; LVMI, left ventricular mass index; E, peak early mitral inflow velocity; e’, peak early diastolic mitral annular velocity.

(A)


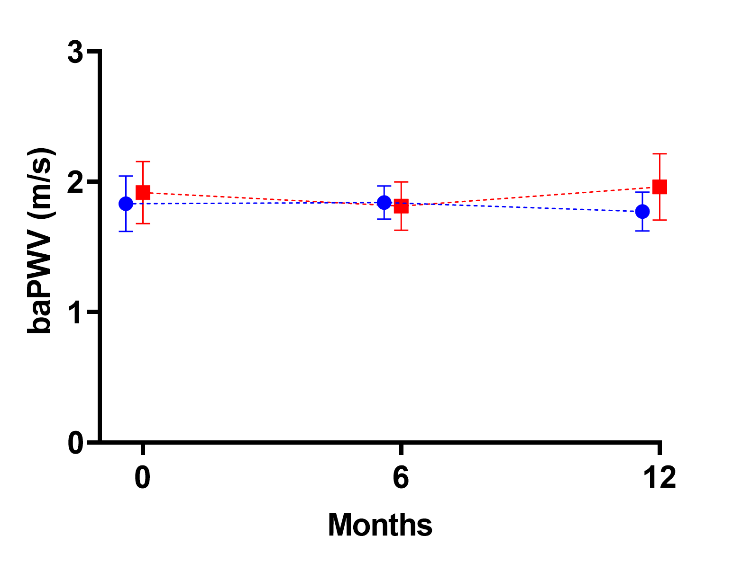


(B)


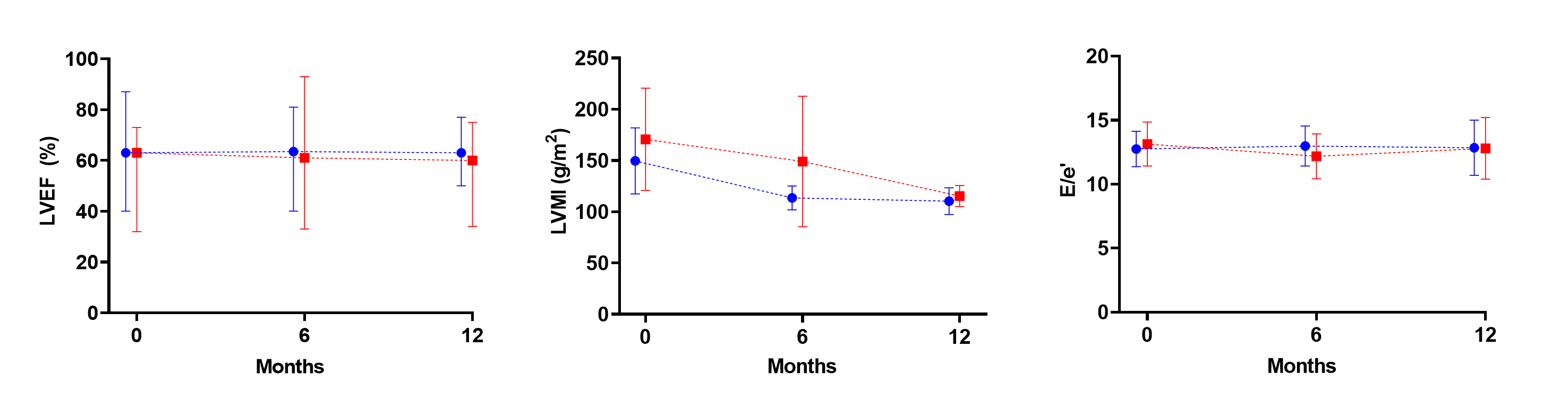


(C)


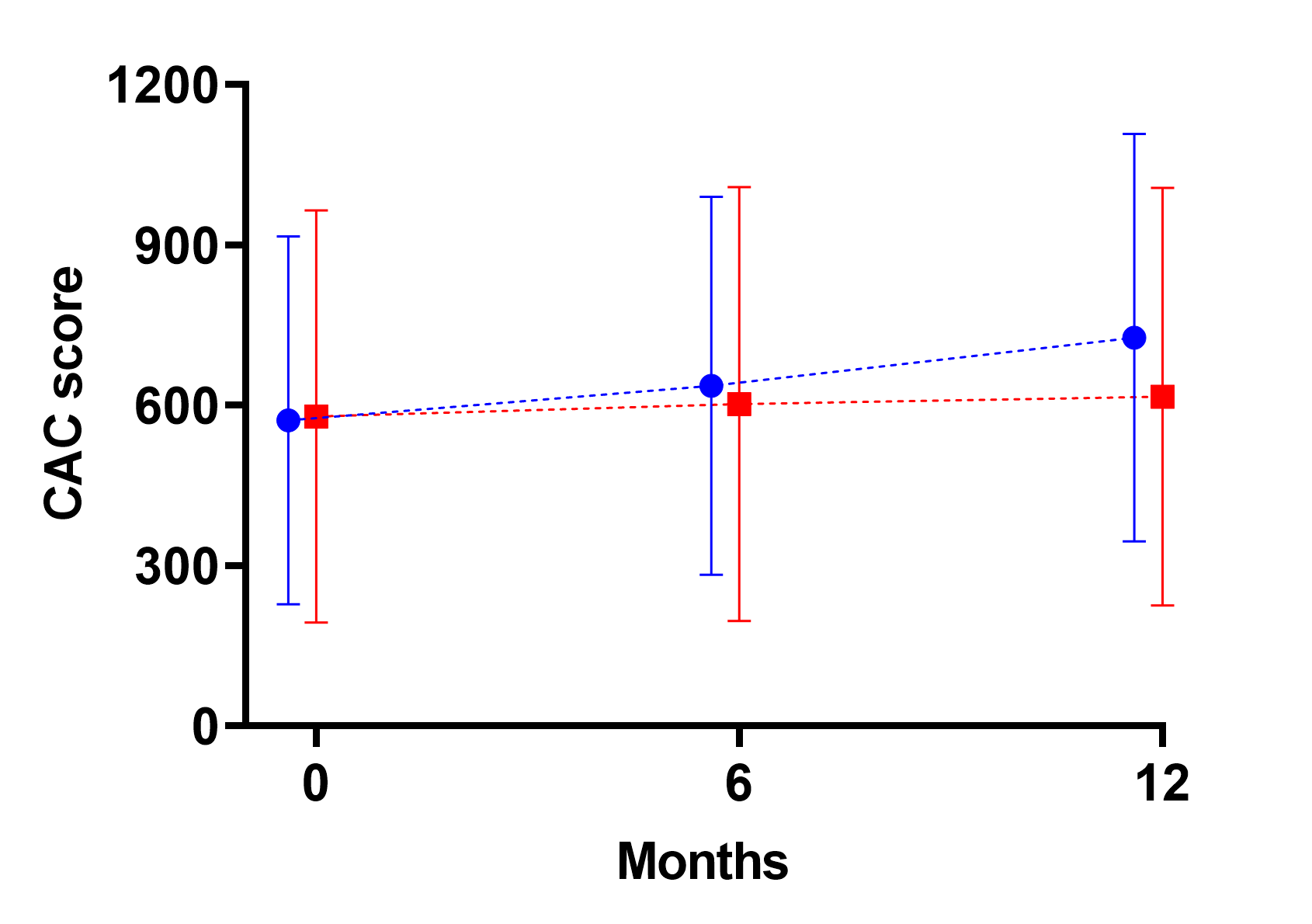

Supplement: Supplementary file 1 — Supplementary Information. [file 41598_2021_90311_MOESM1_ESM.docx]
